# Mental imagery boosts music compositional creativity

**DOI:** 10.1371/journal.pone.0174009

**Published:** 2017-03-15

**Authors:** Sarah Shi Hui Wong, Stephen Wee Hun Lim

**Affiliations:** Department of Psychology, National University of Singapore, Singapore; University of Zurich, SWITZERLAND

## Abstract

We empirically investigated the effect of mental imagery on young children’s music compositional creativity. Children aged 5 to 8 years participated in two music composition sessions. In the control session, participants based their composition on a motif that they had created using a sequence of letter names. In the mental imagery session, participants were given a picture of an animal and instructed to imagine the animal’s sounds and movements, before incorporating what they had imagined into their composition. Six expert judges independently rated all music compositions on creativity based on subjective criteria (consensual assessment). Reliability analyses indicated that the expert judges demonstrated a high level of agreement in their ratings. The mental imagery compositions received significantly higher creativity ratings by the expert judges than did the control compositions. These results provide evidence for the effectiveness of mental imagery in enhancing young children’s music compositional creativity.

## Introduction

Creativity has long been recognised to be intimately linked to acts of creation and innovation across a wide range of domains such as scientific research and discovery, art and, of particular interest to the present research, music composition. Eminent composers such as Mozart and Beethoven have been hailed as “geniuses”, a term first used in the 18^th^ century to describe individuals who displayed remarkable imaginative powers and creative ability [1–2]. Indeed, inventing one’s own music is regarded as a defining hallmark of creative musical thinking [3–4], and a key learning objective in the American National Standards [5], the British National Curriculum [6], and Singapore’s General Music Programme syllabus [7].

### Creativity in music composition

According to the “Four Ps” model of creativity [8–11], conceptualisations of creativity require consideration of four components: the creative person, the creative press (or environment), the creative process, and the creative product. Applied in the context of music composition, a composer’s creativity can be said to be guided by individual differences in creative ability (the creative person), as well as environmental factors (the creative press) such as one’s physical surroundings, schooling experiences, and cultural background that may meaningfully interact with the individual to shape creative cognition [12–17]. In addition, music compositional creativity is facilitated by a dynamic interplay between divergent and convergent thinking (the creative process). For instance, in the domain of music composition, whereas divergent thinking is characterised by the exploration of multiple musical possibilities, convergent thinking relates to arriving at a single best solution through analytically manipulating and evaluating various musical alternatives [16–19]. The tangible and intentional result of a creative person who has engaged in creative thinking processes within a creative environment is, then, a creative product that possesses both novelty and appropriateness (effectiveness) [17, 20–23].

Studies of the music compositional sketches of “geniuses” such as Mozart and Beethoven have dispelled the romanticised vision of creative products being conceived in singular, complete form through divine inspiration without the need for revision, while hinting at creative processes that are far less removed from those of ordinary individuals [24–25]. That is, creativity encompasses ordinary improvisational thinking processes that, nevertheless, yield extraordinary creative products [26–27]. Accordingly, the fundamental question of pedagogical interest is not whether a composer is creative or not, but how a composer’s creativity can be nurtured by facilitating the creative process. In particular, the ability to engage in mental imagery has been proposed to be vital for both convergent and divergent musical thinking in music composition [4], pointing towards its potential to enhance creativity by serving as an enabling factor in the creative process.

### Mental imagery and music compositional creativity

Mental imagery involves a “quasi-perceptual experience: experience that significantly resembles perceptual experience (in any sense mode), but which occurs in the absence of appropriate external stimuli for the relevant perception” (p. 209 in [28]). For instance, auditory imagery can be construed as the mind’s ability to internally imagine sounds (or “hear in the head”) despite their physical absence. When composing music, auditory imagery may be used to imagine novel music in the mind’s ear, form predictions about what a music composition will sound like even before notating or playing it on an instrument, and compare one’s mental “ideal” sounds with the notated musical products intended as expressions of those musical ideas [29–32]. Similarly, visual imagery refers to the ability to “see” visual image representations in the mind in the absence of physical stimulation. Although music compositions are aural in result, they are frequently visually encoded in the form of musical scores [30]. Visual imagery may thus come into play when, for example, composers mentally use traditional or graphic notation symbols to engage in divergent experimentation with the visual representations of sound objects, such as sketching melodic contours and the duration of pitches in the mind’s eye [30].

While there may be modality-specific differences between our internal representations of visual and auditory stimuli [33], a single mental image may incorporate multisensory content. For instance, audiated sound images may not be solely auditory in nature, but may also contain visual information [34]. In interviews with eminent orchestral brass players, Trusheim [35] reported that some of them visualised their ideal sound in association with colours, while one player used the image of a road map to visually represent the structure of a musical piece. Likewise, composers may “translate” mentally visualised scenes and images to auditory form [30, 36]. Building on the view that the art of composition can be compared to the “art of creating illusions” (p. 271), Mountain [30] has pointed out that composers often draw on visual and auditory imagery that refer to elements of the physical world to produce convincing “illusions” in their compositions. For example, Wagner was known to experience persistent visual imagery that accompanied his auditory imagery, whereby he simultaneously visualised scenes and characters for his operas when audiating his composed music for them [37]. Evidently, then, composers more often use visual and auditory imagery in combination, rather than in isolation [30]. As such, the present study focuses on the relationship between visual-auditory (multimodal) mental imagery and music compositional creativity.

Given that creativity has been intimately linked to the “imagination” or one’s faculty of forming mental images of objects that are not physically present [28, 38–39], it is conceivable that the use of mental imagery in music composition may facilitate creativity. Notably, imagery has been posited to aid the identification of non-obvious links and interactions between distant realities due to its perceptual richness and relation to external sources in the physical world [40]. In Rzewski’s *Winnsboro Cotton Mill Blues*, for example, the composer draws on visual and auditory imagery of a cotton mill to make parallels to the hammer mechanism of a piano, as well as the pianist’s mechanical hand and arm movements required for both a sonic representation of the mill’s industrial sounds and a visual-motor representation of repetitive physical labour. Indeed, one can easily identify a rich variety of extra-musical sources of composing inspiration that have lent themselves to visual and auditory imagery in programmatic music [41]. For instance, Saint-Saëns’s *The Carnival of the Animals* conjures a “zoological fantasy” with the auditory sounds of a lion’s majestic roaring, a cuckoo’s gentle calling, and even the braying of donkeys. Similarly, Schubert’s *The Trout* piano quintet uses joyful, rippling arpeggios to recreate the vivid visual image of a fish’s lively movements in sparkling waters as it evades capture by a fisherman. As these examples illustrate, imagery may support the divergent exploration and discovery of novel, meaningful cross-modal associations between visual and auditory stimuli, as well as the development of subsequent creative musical ideas. Here, a fruitful parallel can be drawn to the literary domain, in which compelling colour-sound correspondence and visual-auditory metaphors (e.g., Keats’s *Ode to a Nightingale* refers to “some melodious plot / Of beechen green” [42]) have been recognised to produce creative outcomes [43].

Accordingly, imagery could be highly useful in stimulating inspiration during the generation and exploration of musical ideas in the creative process, whereby composers consider various possibilities and engage in divergent thinking. In consonance with this notion, Antonietti [44] has discovered that mentally representing a problem situation through visual imagery facilitates the generation of a creative solution by allowing one to effectively transform the situation in an unusual way and overcome cognitive obstacles such as functional fixedness.

Furthermore, our mental representations are thought to be flexible and convertible, and thus amenable to manipulation [44]. While some researchers have questioned if visually imaged objects can be ambiguous or reinterpreted [45], more recent work has demonstrated that reconstruals and alternate interpretations of such mental images occur more commonly than previously assumed [46–48]. For instance, Mast and Kosslyn [47] found that perceivers, particularly those with higher spatial transformation abilities, were able to reinterpret ambiguous mentally visualised stimuli when given partial stimulus cues. Studies of auditory imagery also dovetail with those of visual imagery in revealing that auditory images can be manipulated. For example, research by Zatorre, Halpern, and Bouffard [49] has provided behavioural and fMRI evidence for musicians’ use of mental transformation when mentally reversing the notes of familiar melodies in temporal order to judge if presented tunes were exact or inexact reversals of the originals. Moreover, this auditory backward scanning task (transforming auditory images in time) was found to produce similar patterns of cortical activation in the intraparietal sulcus as visual mental rotation tasks (transforming visual images in space) [34, 49–50].

Taken together, it is possible that the manipulability of mental images may aid the creative synthesis of simple components into a useful object [51]. In support of this notion, mental imagery training has been found to induce greater dynamism and flexibility with which children synthesise original mental figures [52]. Hence, to the extent that music composition similarly involves problem-solving and the integration of various musical elements [53–55], one may reasonably expect that the creative benefits of engaging in mental imagery would extend to the context of composing, whereby imagery may facilitate composers’ evaluation, reorganisation, and combination of musical material via mental synthesis to create a creative composition [56].

Indeed, the capacity to engage in visual and auditory imagery has been considered a component of creative musical aptitude in Webster’s [19] Measure of Creative Thinking in Music–Version II (MCTM–II) for young children. For instance, an application task in the MCTM–II involves creating “frog music” based on the visual image of a frog’s movements by hopping and rolling a ball on a piano, while another task requires the children to sing through a microphone based on the auditory image of a robot singing in the shower. The children’s improvisatory performances are then scored on originality (uniqueness of manipulation), flexibility (range of manipulation), syntax (musical appropriateness), and extensiveness (length of a musical response) to evaluate creative musical thinking [19]. According to this line of reasoning, enhancing one’s capacity to engage in visual-auditory mental imagery would foster musical creativity.

### The quest for empirical evidence

Despite the theoretical utility of mental imagery and its plausible conceptual association with music compositional creativity, few studies to date have examined this relationship empirically. As Hubbard [57] has pointed out in his extensive review of auditory imagery literature, empirical research on the use of auditory imagery in music composition is surprisingly “almost non-existent” (p. 316). While the topic of imagery has been studied in music education research as an instructional technique to elicit expressive musical performance [58–59], it has garnered significantly less attention in the domain of music composition.

Although some studies have investigated composers’ use of mental imagery, these works often bear indirect links to compositional creativity as a key outcome of interest. For instance, while Kratus [60] explored the relationship between children’s audiation and their compositions’ specific musical characteristics such as tonal and metric cohesiveness, the compositions’ global creativity was not assessed. Moreover, music audiation was investigated as an innate ability rather than manipulated in direct relation to the composition task at hand. In another study by Adachi and Chino [61], Japanese sophomore and junior undergraduate students were instructed to use cross-modal images of spring such as the sound of melting snow or the movements of falling cherry petals to compose an original sound piece. The students then engaged in divergent thinking to metaphorically match their mental images with sounds expressed through the use of ordinary household items. For example, some student composers conveyed the sound of flying birds’ wings by shaking small plastic-wrapped paper pocket tissues, while others communicated the sound of a spring breeze through waving aluminum foil. Although this study affirms that the use of mental imagery in music composition can be manipulated through explicit instructions to imagine stimuli from one’s physical environment, and that the use of such a technique can stimulate creative composing behaviour, it is less evident if such facilitating effects would otherwise not have arisen in a composing context without cues to engage in mental imagery. Moreover, while Adachi and Chino’s [61] research hints at the creative benefits of using imagery to compose sounds within musically free frames, it remains unresolved if these benefits extend to musically conventional frames when composing in conformance to structural standards, particularly since creativity simultaneously demands appropriateness in addition to novelty. In view of these gaps in extant literature, it is pertinent to experimentally investigate the relationship between the use of mental imagery and music compositional creativity.

Drawing on historical case studies of composers such as Saint-Saëns, we manipulated mental imagery use through instructing young child composers to imagine both the movements (visual imagery) and sounds (auditory imagery) of a given animal, before incorporating what they had imagined into their composition. To measure creativity, we adopted Amabile’s [62] Consensual Assessment Technique (CAT) that has been well-validated across various cultures, creative products, and domains including music composition, creative writing, and drawing [63–65]. The CAT embraces the subjective and socially defined nature of evaluating creative products, given that assessments of creativity are necessarily informed by the judges’ encultured views and personal standards [66]. That is, the CAT concedes Torrance’s [67] claim that creativity “defies precise definition” (p. 43), but rests on the notion that a creative *product* can be evaluated based on consensual criteria in relation to a pool of artefacts being judged, since experts who possess domain knowledge and experience can reliably recognise creativity and arrive at a reasonable level of agreement in their subjective assessments of creativity even without the imposition of any external criteria. Under this view, a product is creative to the extent that the appropriate judges independently agree that it is creative. Accordingly, creativity in this study was operationalised as a novel and effective music composition as evaluated by expert judges using Amabile’s [62] CAT, in line with the bipartite view of creativity as a novel-useful product that has been commonly adopted in current creativity research [8, 12, 68–69]. We hypothesised that the use of visual-auditory mental imagery in young children’s compositional processes would lead to higher creativity ratings of the resulting music compositions.

## Method

### Participants

The participants were 9 children (6 were girls) enrolled in a special group-based music educational programme in Singapore (Music for Young Children; MYC, Newton Branch), although one participant’s data were subsequently excluded from analyses due to non-conformance to instructions. Participants were 5- to 8-year-olds (*M* = 6.85, *SD* = 1.36). As the MYC programme integrates music composition in its curriculum along with keyboard performance, all participants possessed prior experience in composition and had also received similar training related to music notation and basic compositional techniques. Participants were recruited after written informed consent from their parents and the music school’s Principal had been obtained. This study’s protocol was approved by the Institutional Review Board of the National University of Singapore.

### Pilot study procedure

To select the imagery stimuli for the main study on the basis of their high auditory and visual imageability, a pilot study was conducted with 12 younger children between the ages of 3 to 5 years who were enrolled in the same music programme, but who did not participate in the main study. If these younger children were able to imagine the imagery stimuli with relative ease, then the older participants would likely be able to do so as well, if not actually with greater ease.

The children who participated in the pilot study reported how easy it was for them to imagine a given animal moving, as well as to imagine the sounds produced by the animal. Six animals were evaluated: *bee*, *cat*, *frog*, *horse*, *snake*, and *tiger*. To ensure that participants fully understood the task requirements, two objects that presumably cannot move or produce sounds on their own accord (*flower* and *snowman*) were also included as check items. Participants rated the eight imagery objects’ imageability on a scale from 1 (*not easy at all*) to 5 (*very*, *very easy*). The scale points were represented by black dots that increased in size by a constant ratio of 1:1.5 to aid participants in understanding the continuum.

Participants’ auditory and visual imageability ratings were collapsed to yield a mean imageability score for each animal. Data from four participants who did not fully understand the task, as indicated by their high mean imageability ratings above the mid-point of the scale for at least one of the two check items, were excluded from analyses. The three animals with the highest imageability scores were: *bee* (*M* = 4.94, *SD* = 0.18), *frog* (*M* = 4.94, *SD* = 0.18), and *tiger* (*M* = 5.00, *SD* = 0.00). Accordingly, these animals served as the critical imagery items in the main study.

### Main study procedure

The main study consisted of two phases: a composition phase, and a judging phase.

#### Composition phase

Each participant underwent two music composition sessions comprising the mental imagery condition and control condition, respectively. Both sessions were conducted at least a week apart in counterbalanced order. Each session was conducted in groups of three to six participants, and lasted approximately 30 minutes. During the sessions, participants were allowed to freely try out their compositions on digital pianos. They were also given headphones to ensure that they could compose independently without external influence, and that they could not hear their peers’ compositions.

In the control condition, participants were presented with the letter names of the seven white keys of a piano keyboard (from A through to G), and were instructed to write down any six letter names. Participants were told that this sequence of letters would constitute the motif of their composition, and that the letters could be used more than once. Following which, participants were asked to compose an 8-bar melody that sounded good to them based on the temporary motif that they had created, which could be modified in the course of composing. This compositional task was designed with reference to a traditional approach towards music composition, whereby composers create melodies through generating and exploring musical material based on a short motif (e.g., the hallmark four-note opening motif of Beethoven’s Fifth Symphony) that lends itself to development [70]. This technique of motif development has been previously documented as being spontaneously used by child composers [71–72].

In the mental imagery condition, participants were randomly assigned one of the three animals that had been selected via the pilot study. Participants were given handouts containing a picture of their assigned animal in a stationary position, and instructions for engaging in visual-auditory (multimodal) mental imagery. Specifically, participants were asked to “hear” and imagine in their head the sounds made by their given animal, as well as to “see” and imagine the given animal moving. Following which, participants were instructed to compose an 8-bar melody that sounded good to them based on the animal they had imagined, such that listeners would be able to hear the animal “come alive” when listening to the composition. Imagery instructions were verbally reinforced for each individual participant to ensure understanding.

After each composition session, all participants reported how easy it was for them to compose the piece of music, and how creative they perceived their composition to be. All ratings were performed on a 5-point scale, with points represented by black dots that increased in size by a constant ratio of 1:1.5 to illustrate the continuum. Participants were also asked to give their music composition a title, and to describe their composition. Responses to these check items were analysed to ensure that participants fully understood the imagery instructions given to them, and that their compositions were indeed about the animals that they had been told to imagine. With the exception of one participant who was subsequently excluded from analyses, all remaining 8 participants reported that they had adhered to the imagery instructions, and had written their imagery composition based on the sounds and movements of their assigned animal.

#### Judging phase

Participants’ compositions were entered into a music notation software (Sibelius 6), and piano sound recordings were generated for each composition via the software’s playback function. The musical stimuli were then presented via a Microsoft Powerpoint slideshow to six expert judges. In accordance to Amabile’s [62] recommendation that judges be knowledgeable about the domain in question and possess domain-related experience, all judges were recruited on the basis of their background as instrumental music teachers. The judges also possessed prior experience in teaching young children either in MYC or private music lessons, in line with Hickey’s [65] finding that the most reliable judges to assess the creativity of children’s musical compositions are the music teachers who teach the children themselves or who have extensive experience teaching children.

The judges independently listened to and then rated each composition’s creativity (“Using your own subjective definition of creativity, how creative was this composition?”) on a scale from 1 (*not at all*) to 7 (*extremely*). This item had been constructed based on Amabile’s [62] CAT, as well as Webster and Hickey’s [73] finding that *global* (consideration of broader issues rather than specific musical characteristics) and *implicit* (deliberately little description of the rated dimension and criteria to consider) rating scales are best predictive for the construct of creativity. Following Amabile’s [62] guidelines, the judges were instructed to rate the compositions relative to one another, rather than against some absolute standard. The judges were further encouraged to make use of the entire range of the 7-point scale. Each judge listened to the compositions in a different random order, so as to eliminate the influences of method artefacts on interjudge consistency. To avoid demand characteristics and strengthen construct validity, the judges were not presented with the titles and descriptions that the child composers had given their compositions. This prevented any speculation of the study’s hypothesis, and also ensured that the judges would not rate a composition more favourably simply because they perceived its description to be a creative one or because the composition matched their musical expectations of its intended programmatic content. Instead, the judges’ evaluations were based primarily on the inherent musical qualities of the compositions.

## Results

### Manipulation check

To check that task difficulty was comparable between the mental imagery and control conditions, a paired samples *t*-test was performed comparing the child composers’ ratings of perceived ease in composing both music compositions. No significant difference in reported ease of composition was found between the mental imagery (*M* = 3.75, *SD* = 1.04) and control (*M* = 3.25, *SD* = 0.89) conditions, *t*(7) = 1.18, *p* = .28. Participants did not perceive either composition task to be significantly easier or more difficult.

### Main analyses

Cronbach’s alpha was computed to test interjudge reliability, whereby a figure of .70 or higher was taken to indicate an acceptable level of agreement among the judges [74]. The six expert judges demonstrated good internal consistency in their ratings of creativity (α = .81). Mean creativity ratings for each music composition were then computed by averaging all judges’ ratings. These mean ratings were used in subsequent analyses.

Consistent with our hypothesis, a paired samples directional *t*-test revealed that the expert judges awarded significantly higher creativity ratings to the mental imagery compositions (*M* = 4.52, *SD* = 1.26) than to the control compositions (*M* = 3.71, *SD* = 0.69), *t*(7) = 2.30, *p* = .027, one-tailed, *d* = 0.81.

**Fig 1:**
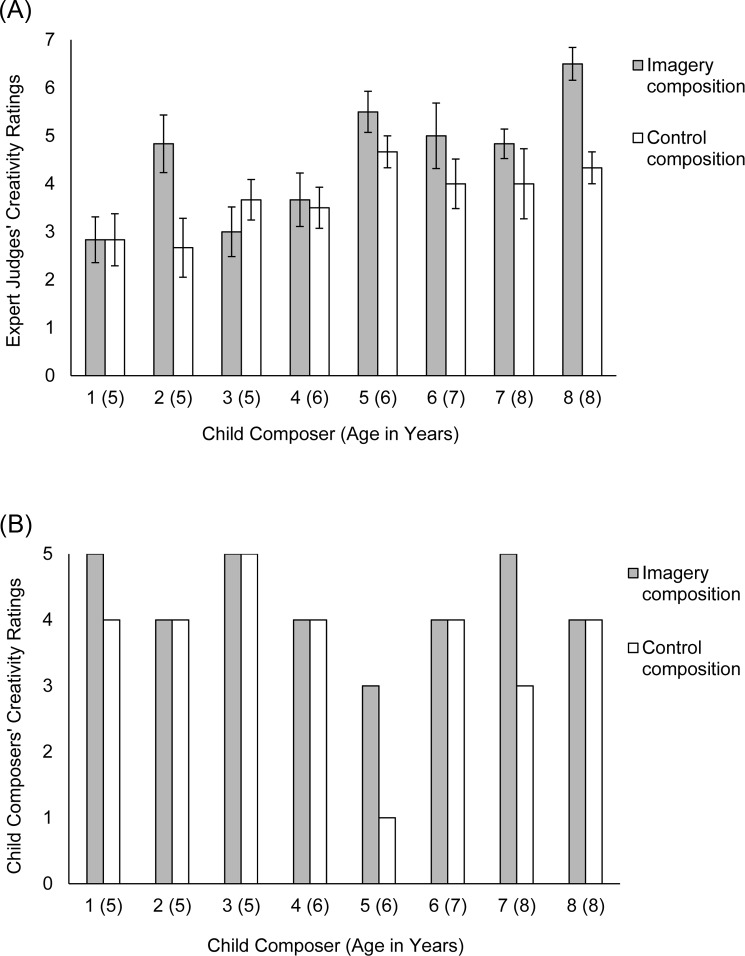
Creativity ratings of individual child composers’ imagery and control compositions. (A) Expert judges’ (*N* = 6) ratings, on a 7-point scale, of each child composer’s imagery and control compositions. Error bars represent standard errors. (B) Individual child composers’ ratings, on a 5-point scale, of their own imagery and control compositions.

Echoing the expert judges’ ratings, a paired-samples directional *t*-test indicated that the child composers perceived their own mental imagery compositions to be significantly more creative (*M* = 4.25, *SD* = 0.71) than their control compositions (*M* = 3.63, *SD* = 1.19), *t*(7) = 1.93, *p* = .048, one-tailed, *d* = 0.68, although the child composers’ creativity ratings did not significantly correlate with those by the expert judges, *r*(16) = -.38, *p* = .14. The child composers’ ratings of their compositions are presented in Fig 1 alongside the expert judges’ ratings.

## Discussion

We investigated the use of visual-auditory mental imagery in young children’s music compositional processes, and established empirical support for its positive effects on music compositional creativity. As assessed by a panel of expert judges using Amabile’s [62] CAT, the same child composers produced more creative music compositions when they engaged in mental imagery. Thus, in line with existing conceptualisations of creativity as a mental process that is responsive to enabling conditions [4], our results affirm that creativity in music composition can be enhanced by the use of cognitive techniques such as mental imagery. To the best of our knowledge, the present study is the first systematic attempt to date to provide experimental evidence for the benefits of mental imagery on young children’s music compositional creativity.

Although some indirect support for the creative benefits of mental imagery has previously been accrued in meta-analytic research and correlational studies that examined the relationship between visual imagery abilities and divergent thinking performance [75–77], it must be noted that divergent thinking alone is insufficient for music compositional creativity. That is, simply exploring a large volume of original but merely bizarre musical ideas is inadequate if convergent thinking is not employed to critically evaluate, revise, and refine these initial musical thoughts towards forming a cohesive musical composition [17, 72]. Focusing on creative musical products as manifestations of the interaction between the child composers’ divergent and convergent thinking processes thus allowed for a more holistic investigation of music compositional creativity in our study. Moreover, through examining the effects of mental imagery in the domain of music composition—a form of musical expression and meaning-making that both educators and researchers consider valuable and integral to musical learning [78–79]—this study fills a knowledge lacuna in current research that has hitherto remained relatively unexplored as compared to related domains such as music performance [58–59].

At the same time, our findings corroborate Piagetian developmental theories that children as young as 5 years of age (preoperational stage of development) are capable of engaging in mental imagery, and are able to use their mental images to form symbolic representations in the absence of actual perception [80–81]. According to Bruner [82], children represent images in three modes: the enactive mode (representing past events through motor actions), iconic mode (selectively organising images of perceptual events and objects), and symbolic mode (transforming experiences into more abstract representations such as words). In the present study, it appears that the child composers (5- to 8-year-olds) were able to symbolically transform their visual-auditory mental imagery of an animal’s sounds and movements into musical symbols to create a more creative product than when they had composed by generating a musical motif without explicit instructions to engage in mental imagery.

Furthermore, the creative advantage of employing mental imagery emerged even though the children rated the imagery composition task as being of similar difficulty as the control composition task, with mean ratings around the midpoint of the scale. That both tasks were perceived to be of comparable difficulty (or ease) is, perhaps, unsurprising from a developmental perspective. Just as how the use of mental imagery has been reported to be evident even in preschoolers’ early make-believe play [83], the traditional compositional approach of motif development in the control task has been spontaneously adopted by children in other prior studies [71–72]. In addition, both tasks similarly involved composing based on concrete stimuli—an animal vs. letter names corresponding to the white keys of a piano keyboard—that the children possessed prior knowledge of. Yet, the use of mental imagery significantly boosted music compositional creativity, accentuating the utility of this cognitive technique.

### Practical implications

These results have promising implications for music composition pedagogy, whereby music educators can incorporate mental imagery techniques in their teaching of composition to nurture their students’ creativity. A particular strength of the mental imagery strategy is that it appears to be relatively easy to implement in the classroom—the child composers in the present sample profited from mental imagery even without training prior to completing the experimental task, with the benefits of mental imagery for their creative performance emerging immediately. As younger children often tend not to spontaneously use mental imagery—particularly visual imagery—in problem-solving although they are capable of doing so [84–85], they may initially require explicit instructions or cues to engage in mental imagery when solving creative compositional problems such as identifying musical possibilities and making aesthetic judgments [84].

With experience and the development of domain-relevant skills, knowledge, and expertise over time, composers may become more proficient at mentally manipulating and organising musical material through the use of imagery, and may also be more adept at applying diverse compositional techniques to effectively “translate” their extra-musical images and fully express their creative ideas [30, 86]. For instance, while the mental imagery of children in the preoperational stage (ages 2–7) is assumed to be “static” in that it is limited to representing unchanging states of objects and events [80–81], developmentally more mature children are capable of flexibly transforming their mental imagery, such as mentally re-experiencing and transforming a melody to identify an absent melodic stimulus in a transposed sequence of the original melody [87]. Moreover, although imposed (other-generated) imagery has been postulated to be more effective than fully induced (self-generated) imagery for younger children [88–89], older or more musically experienced children can potentially be encouraged to freely draw upon images of their choice in an autonomous way. As Trusheim [35] has suggested, mental imagery skills can be developed through practice and application, especially in terms of vividness (clarity of the images), controllability (one’s ability to manipulate the images), and fluency (number of images generated and recalled). Indeed, music training has been found to enhance auditory imagery, presumably due to more efficient imagery processing in auditory cortical areas [90].

### Future directions

This study represents an initial foray into the relationship between the use of mental imagery and music compositional creativity, targeting a select group of young students who have received music compositional training under the special MYC programme. One limitation of this study is hence its small sample, although it is worth noting that we observed medium to large effect sizes in spite of this. Further research could validate and extend our findings by involving larger samples of general music learners and composers.

In particular, future work can investigate and identify the specific ways in which young children use mental imagery to enhance music compositional creativity. Although the child composers received similar instructions to imagine the sounds and movements of a given animal, each individual may have applied the resulting mental images in a different way, or during a different stage in the compositional process. For instance, whereas some children in this study attempted to directly translate their mental images into musical contexts (e.g., expressing a frog’s croaking via the use of dissonant chords), others seemed to use their images as a broader concept or mood that shaped their final compositional product (e.g., expressing a tiger’s movements via creating a suspenseful mood). Subsequent studies can thus qualitatively probe young children’s music compositional processes to gain deeper insight about the nature of their mental imagery and the ways in which it is applied, as well as map the developmental trajectory of imagery use in music composition in view of age-related differences in image generation and maintenance abilities [85].

At the same time, future studies can extend the effects of mental imagery on music compositional creativity to more diverse imagery targets besides the animals used in the present research. In our pilot study, some children reported high imageability for a *flower* and *snowman* even though these two objects presumably could not move or produce sounds on their own accord. Upon consideration of young children’s ability to engage in highly imaginative make-believe play [83], exploring the parameters of stimuli that boost child composers’ music compositional creativity may constitute an intriguing possibility.

Furthermore, given the prevalence of multisensory imagery in everyday cognition [91–92], it may be useful to investigate the effects of different modalities of mental imagery on music compositional creativity beyond this study’s focus on visual-auditory imagery. Notably, Hubbard [34] has reviewed evidence suggesting that auditory imagery may evoke kinaesthetic information related to speech articulation and motor movements in dance and imaged musical performance, in addition to visual information. That is, there may be overlaps amongst auditory, visual, and kinaesthetic imagery. For instance, while mentally “seeing” an external object (an animal) perform movements more likely involved primarily visual imagery for the child composers in this study [93], kinaesthetic or motor imagery may also occur if individuals simultaneously imagine themselves performing actions such as hopping like a frog (although none of the children in this study reported having done so). Accordingly, future work may seek to distinguish the effects and relative contributions of each imagery modality to music compositional creativity by inducing them independently and in combination. It is possible that engaging in unimodal imagery that does not allow one to move freely between imagery modalities may hamper music compositional creativity, if doing so creates a task constraint that interferes with creative freedom and decision-making [72], or if the unimodal images generated are impoverished. As Mountain [30] suggests, multimodal images serve as more complete models that possess the characteristics of an entity in our physical world, and are likely richer and more vivid than unimodal images. Hence, to the extent that our perception has evolved as a means of understanding our physical environment, it may be that our perception of music is conditioned by our knowledge of multisensory stimuli around us [30], such that music compositions based on multimodal images that produce more complete or vivid illusions are more likely to be perceived as being effectively novel, as compared to compositions written using unimodal imagery.

Relatedly, the qualities associated with music compositions that are perceived as “creative” are not readily inferred from the present study, since the CAT crucially depends on subjective global evaluations of creativity and does not ask raters to justify their ratings [62]. Our analyses revealed a discrepancy between what the expert judges and child composers perceived as creative. Specifically, the children’s creativity ratings of their own compositions did not correlate with those awarded by the expert judges, even though the children similarly rated their imagery composition as being significantly more creative than their control composition. As such, future research could probe children’s conceptualisation of creativity, such that music educators may be better able to understand children’s perspective of their musical efforts, and effectively teach creative music composition.

In addition, since this study has established that music compositional creativity can be promoted through the use of mental imagery as a cognitive strategy, a meaningful avenue for future work is to explore more techniques to increase young children’s repertoire of approaches towards creatively composing music. For instance, some studies with seventh-and ninth-grade students have demonstrated that music compositions that had been notated using non-traditional graphic patterns were rated as aurally more creative by expert judges, as compared to compositions that had been written using traditional staff notation [94–96]. Presumably, the use of graphic notations provided a relatively more unrestricted platform for the representation and invention of new types of sounds [96], thereby encouraging the adoption of more diverse music compositional strategies that stimulated divergent thinking and subsequently enhanced creativity [95]. Considered alongside the present study, we hope that these findings that creative thinking in music composition can be developed may encourage further empirical work in this direction.

## Conclusion

While musical creativity has often been associated with “geniuses” such as Mozart and Beethoven, this research demystifies creativity by providing suggestive evidence that it can potentially be nurtured in ordinary individuals through essentially ordinary cognitive processes that yield extraordinary products [27]. The use of mental imagery appears to be one such “ordinary” cognitive process that promotes music compositional creativity.

## Supporting information

S1 Dataset Main Study Data. (XLSX)
